# A systematic review of progranulin concentrations in biofluids in over 7,000 people—assessing the pathogenicity of *GRN* mutations and other influencing factors

**DOI:** 10.1186/s13195-024-01420-z

**Published:** 2024-03-28

**Authors:** Imogen J. Swift, Rosa Rademakers, NiCole Finch, Matt Baker, Roberta Ghidoni, Luisa Benussi, Giuliano Binetti, Giacomina Rossi, Matthis Synofzik, Carlo Wilke, David Mengel, Caroline Graff, Leonel T. Takada, Raquel Sánchez-Valle, Anna Antonell, Daniela Galimberti, Chiara Fenoglio, Maria Serpente, Marina Arcaro, Stefanie Schreiber, Stefan Vielhaber, Philipp Arndt, Isabel Santana, Maria Rosario Almeida, Fermín Moreno, Myriam Barandiaran, Alazne Gabilondo, Johannes Stubert, Estrella Gómez-Tortosa, Pablo Agüero, M. José Sainz, Tomohito Gohda, Maki Murakoshi, Nozomu Kamei, Sarah Kittel-Schneider, Andreas Reif, Johannes Weigl, Jinlong Jian, Chuanju Liu, Ginette Serrero, Thomas Greither, Gerit Theil, Ebba Lohmann, Stefano Gazzina, Silvia Bagnoli, Giovanni Coppola, Amalia Bruni, Mirja Quante, Wieland Kiess, Andreas Hiemisch, Anne Jurkutat, Matthew S. Block, Aaron M. Carlson, Geir Bråthen, Sigrid Botne Sando, Gøril Rolfseng Grøntvedt, Camilla Lauridsen, Amanda Heslegrave, Carolin Heller, Emily Abel, Alba Gómez-Núñez, Roger Puey, Andrea Arighi, Enmanuela Rotondo, Lize C. Jiskoot, Lieke H. H. Meeter, João Durães, Marisa Lima, Miguel Tábuas-Pereira, João Lemos, Bradley Boeve, Ronald C. Petersen, Dennis W. Dickson, Neill R. Graff-Radford, Isabelle LeBer, Leila Sellami, Foudil Lamari, Fabienne Clot, Barbara Borroni, Valentina Cantoni, Jasmine Rivolta, Alberto Lleó, Juan Fortea, Daniel Alcolea, Ignacio Illán-Gala, Lucie Andres-Cerezo, Philip Van Damme, Jordi Clarimon, Petra Steinacker, Emily Feneberg, Markus Otto, Emma L. van der Ende, John C. van Swieten, Harro Seelaar, Henrik Zetterberg, Aitana Sogorb-Esteve, Jonathan D. Rohrer

**Affiliations:** 1 Department of Neurodegenerative Disease, Dementia Research Institute, UCL Institute of Neurology, Queen Square, London, UK; 2 Department of Neurodegenerative Disease, Dementia Research Centre, UCL Institute of Neurology, Queen Square, London, WC1N 3BG UK; 3 Department of Neuroscience, Mayo Clinic, Jacksonville, FL USA; 4 VIB Center for Molecular Neurology, VIB, Antwerp, Belgium; 5 Department of Biomedical Sciences, University of Antwerp, Antwerp, Belgium; 6 Molecular Markers Laboratory, IRCCS Istituto Centro San Giovanni Di Dio Fatebenefratelli, Brescia, Italy; 7 MAC-Memory Clinic and Molecular Markers Laboratory, IRCCS Istituto Centro San Giovanni Di Dio Fatebenefratelli, Brescia, Italy; 8 Unit of Neurology V and Neuropathology, Fondazione IRCCS Istituto Neurologico Carlo Besta, Milan, Italy; 9 Division Translational Genomics of Neurodegenerative Diseases, Hertie-Institute for Clinical Brain Research, University of Tübingen, Tübingen, Germany; 10 German Center of Neurodegenerative Diseases (DZNE), Tübingen, Germany; 11 Department of Neurobiology, Care Sciences and Society, Center for Alzheimer Research, Division of Neurogeriatrics, BioclinicumKarolinska Institutet, Solna, Sweden; 12 Unit for Hereditary Dementias, Theme Inflammation and Aging, Karolinska University Hospital, Solna, Sweden; 13 Department of Neurology, Hospital das Clinicas, University of São Paulo Medical School, São Paulo, Brazil; 14 Alzheimer’s Disease and Other Cognitive Disorders Unit, FRCB-IDIBAPS, Institut de Neurociències, Neurology Service, Hospital Clínic de Barcelona, Universitat de Barcelona (UB), 08036 Barcelona, Spain; 15 Dept. of Biomedical, Surgical and Dental Sciences, University of Milan, Milan, Italy; 16 Neurodegerative Diseases Center, Fondazione IRCCS Ca’ Granda, Ospedale Maggiore Policlinico, Milan, Italy; 17 Department of Neurology, Otto Von Guericke University, Magdeburg, Germany; 18 Neurology Department, Centro Hospitalar E Universitário de Coimbra, Coimbra, Portugal; 19 Center for Neuroscience and Cell Biology, Centre for Innovative Biomedicine and Biotechnology, University of Coimbra, Coimbra, Portugal; 20 Faculty of Medicine, University of Coimbra, Coimbra, Portugal; 21 Cognitive Disorders Unit, Department of Neurology, Donostia University Hospital, San Sebastian, Gipuzkoa Spain; 22 Neuroscience Area, Biodonostia Health Research Insitute, San Sebastian, Gipuzkoa Spain; 23 Department of Obstetrics and Gynecology, Rostock University Medical Center, Rostock, Germany; 24 Department of Neurology, Fundación Jiménez Díaz, Madrid, Spain; 25 Department of Nephrology, Faculty of Medicine, Juntendo University, Tokyo, Japan; 26 Department of Endocrinology and Metabolism, Hiroshima Red Cross Hospital & Atomicbomb Survivors Hospital, Hiroshima, Japan; 27 Institute for Clinical Research, National Hospital Organization, Kure Medical Center and Chugoku Cancer Center, Hiroshima, Japan; 28 Department of Psychiatry, University College Cork, Cork, Ireland; 29 Department of Psychiatry, Psychotherapy and Psychosomatic Medicine, University Hospital Würzburg, Würzburg, Germany; 30 Department of Psychiatry, Psychotherapy and Psychosomatic Medicine, University Hospital Frankfurt, Frankfurt, Germany; 31 Department of Psychiatry, Hospital in Tauberbischofsheim, Tauberbischofsheim, Germany; 32 University of Pennsylvania, Gene Therapy Program, Philadelphia, USA; 33 Department of Orthopaedics & Rehabilitation, Yale University School of Medicine, New Haven, CT USA; 34 A&G Pharmaceutical Inc, Columbia, MD USA; 35 Program in Oncology, University of Maryland Greenebaum Comprehensive Cancer Center, Baltimore, MD USA; 36 Center for Reproductive Medicine and Andrology, Martin Luther University Halle-Wittenberg, Halle, Germany; 37 Department of Urology, Martin Luther University Halle-Wittenberg, Halle, Germany; 38 Department of Neurodegenerative Diseases, Hertie Institute for Clinical Brain Research, University of Tübingen, Tübingen, Germany; 39 DZNE, German Center for Neurodegenerative Diseases, Tübingen, Germany; 40 Department of Neurological and Vision Sciences, Neurophysiology Unit, ASST SpedaliCivili, Brescia, Italy; 41 Department of Neurological and Psychiatric Sciences, University of Florence, Viale Morgagni, 85, 50134 Florence, Italy; 42 Department of Neurology, University of California, Los Angeles, California USA; 43 Department of Psychiatry Semel Institute for Neuroscience and Human Behavior, University of California, Los Angeles, California USA; 44 Regional Neurogenetic Centre, ASPCZ, Lamezia Terme, Italy; 45 Department of Neonatology, Tuebingen University Hospital, Tuebingen, Germany; 46 Leipzig Research Center for Civilization Diseases - LIFE, University of Leipzig, Leipzig, Germany; 47 Hospital for Children and Adolescents, University of Leipzig, Leipzig, Germany; 48 Center of Pediatric Research (CPL), University of Leipzig, Leipzig, Germany; 49 Department of Oncology, Mayo Clinic, Rochester, MN USA; 50 Department of Neurology, University of Colorado Anschutz Medical Campus, Denver, CO USA; 51 Department of Neurology and Clinical Neurophysiology, Trondheim University Hospital, Trondheim, Norway; 52 Department of Neuromedicine and Movement Science, Faculty of Medicine and Health Sciences, NTNU. , Trondheim, Norway; 53 Department of Research, Trondheim University Hospital, Trondheim, Norway; 54 Department of Neurology and Alzheimer Center Erasmus MC, Erasmus MC University Medical Center, Rotterdam, Netherlands; 55 Department of Neurology, Mayo Clinic, Rochester, MN USA; 56 Department of Neurology, Mayo Clinic, Jacksonville, FL USA; 57 Sorbonne UniversitéInserm U1127, CNRS UMR 7225, Institut du Cerveau Et La Moelle Épinière (ICM), AP-HP - Hôpital Pitié-Salpêtrière, Paris, France; 58 Centre de Référence Des Démences Rares Ou Précoces, IM2A, Département de Neurologie, AP-HP - Hôpital Pitié-Salpêtrière, Paris, France; 59 UF de Biochimie Des Maladies Neurométaboliques Et Neurodégénératives, Service de Biochimie Métabolique, AP-HP - Hôpital Pitié-Salpêtrière, Paris, France; 60 UF de Neurogénétique Moléculaire Et Cellulaire, Département de Génétique, AP-HP, Hôpitaux Universitaires La Pitié Salpêtrière-Charles Foix, Paris, France; 61 Department of Clinical and Experimental Sciences, University of Brescia, Brescia, Italy; 62 Neurology Department. Hospital Sant Pau, Memory Unit, Barcelona, Spain; 63 Centro de Investigación Biomédica en Red en Enfermedades Neurodegenerativas (CIBERNED), 28031 Madrid, Spain; 64 Autonomous University of Barcelona, 08913 Barcelona, Spain; 65 Institute of Rheumatology, Na Slupi 4, 12850 Prague 2 Prague, Czech Republic; 66 Laboratory of Neurobiology, Flanders Interuniversity Institute for Biotechnology, Katholieke Universiteit Leuven, Campus Gasthuisberg, 3000 Louvain, Belgium; 67 Centro de Investigación Biomédica en Red Sobre Enfermedades Neurodegenerativas (CIBERNED), Madrid, Spain; 68 Memory Unit, Department of Neurology, Institut d’Investigacions Biomèdiques Sant Pau - Hospital de Sant Pau, Universitat Autònoma de Barcelona, Barcelona, Spain; 69 Department of Neurology, Martin-Luther University Halle-Wittenberg, University Clinic Halle, Halle (Saale), Germany; 70 Department of Neurology, Klinikum Rechts Der Isar, Technical University of Munich, Munich, Germany; 71 UK Dementia Research Institute at University College London, UCL Queen Square Institute of Neurology, University College London, London, UK; 72 Institute of Neuroscience and Physiology, Sahlgrenska Academy at the University of Gothenburg, 43180 Mölndal, Sweden; 73 Department of Psychiatry and Neurochemistry, Institute of Neuroscience and Physiology, Sahlgrenska Academy at the University of Gothenburg, Mölndal, Sweden; 74 Clinical Neurochemistry Laboratory, Sahlgrenska University Hospital, Mölndal, Sweden; 75 Hong Kong Center for Neurodegenerative Diseases, Hong Kong, China

**Keywords:** Frontotemporal dementia, Progranulin

## Abstract

**Background:**

Pathogenic heterozygous mutations in the progranulin gene (*GRN*) are a key cause of frontotemporal dementia (FTD), leading to significantly reduced biofluid concentrations of the progranulin protein (PGRN). This has led to a number of ongoing therapeutic trials aiming to treat this form of FTD by increasing PGRN levels in mutation carriers. However, we currently lack a complete understanding of factors that affect PGRN levels and potential variation in measurement methods. Here, we aimed to address this gap in knowledge by systematically reviewing published literature on biofluid PGRN concentrations.

**Methods:**

Published data including biofluid PGRN concentration, age, sex, diagnosis and *GRN* mutation were collected for 7071 individuals from 75 publications. The majority of analyses (72%) had focused on plasma PGRN concentrations, with many of these (56%) measured with a single assay type (Adipogen) and so the influence of mutation type, age at onset, sex, and diagnosis were investigated in this subset of the data.

**Results:**

We established a plasma PGRN concentration cut-off between pathogenic mutation carriers and non-carriers of 74.8 ng/mL using the Adipogen assay based on 3301 individuals, with a CSF concentration cut-off of 3.43 ng/mL. Plasma PGRN concentration varied by *GRN* mutation type as well as by clinical diagnosis in those without a *GRN* mutation. Plasma PGRN concentration was significantly higher in women than men in *GRN* mutation carriers (*p* = 0.007) with a trend in non-carriers (*p* = 0.062), and there was a significant but weak positive correlation with age in both *GRN* mutation carriers and non-carriers. No significant association was seen with weight or with *TMEM106B* rs1990622 genotype. However, higher plasma PGRN levels were seen in those with the *GRN* rs5848 CC genotype in both *GRN* mutation carriers and non-carriers.

**Conclusions:**

These results further support the usefulness of PGRN concentration for the identification of the large majority of pathogenic mutations in the *GRN* gene. Furthermore, these results highlight the importance of considering additional factors, such as mutation type, sex and age when interpreting PGRN concentrations. This will be particularly important as we enter the era of trials for progranulin-associated FTD.

**Supplementary Information:**

The online version contains supplementary material available at 10.1186/s13195-024-01420-z.

## Introduction

Heterozygous mutations in the progranulin gene (*GRN*) are a key cause of frontotemporal dementia (FTD) [2, 7]. These pathogenic *GRN* variants cause haploinsufficiency, resulting in a significantly decreased concentration of the progranulin protein (PGRN), a reduction which can be measured in biofluids [13, 15]. The discovery of this core pathophysiological process has led to the development of clinical trials that aim to increase the PGRN concentration in these mutation carriers by targeting key mechanisms involved in the biology of PGRN. However, to establish the efficacy of treatment, robust and effective measures of PGRN biofluid concentration are required. In turn, in order to correctly interpret results, a more comprehensive understanding of how PGRN concentrations change with disease and what factors influence such concentrations is required.

Despite multiple studies now being published on PGRN concentration, a number of questions remain. Firstly, what level signifies the presence of a pathogenic mutation? Although significantly reduced PGRN levels in mutation carriers occur compared with non-carriers, previous studies have shown some overlap between these groups, with debate on the best cut-off value to define pathogenicity: proposed plasma cut-offs have ranged from 61.5 ng/mL to 112.0 ng/mL [9, 14]. Secondly, what factors affect this variability in PGRN concentration? Little is known about the effect of pre-analytical or processing factors but existing literature indicates that different types of *GRN* mutation may cause lower or higher PGRN concentrations [22, 24, 28]. Moreover, previous studies have highlighted the influence of certain genetic polymorphisms, including *TMEM106B* rs1990622 and *GRN* rs5848, on PGRN concentration [10, 16, 25]. Finally, several studies have highlighted the influence of different clinical phenotypes and biological sex on PGRN concentration [1, 10, 30].

In this study, we aimed to explore these questions using a retrospective analysis of published data on biofluid PGRN concentration.

## Methods and demographics

### Data acquisition

We initially undertook a PubMed search for all publications reporting human biofluid PGRN concentrations in either disease or healthy controls (Supplementary Fig. 1). 154 publications were identified (up to a cut-off search date of January 2020): data was available online for 12 of these and for the further 142 publications, corresponding authors were contacted directly to enquire about the availability of data. As well as the specific PGRN concentration, the following data were requested: the assay that was used, whether a *GRN* mutation was present (and if so, which one), symptomatic vs presymptomatic status (if *GRN* mutation present), clinical diagnosis (e.g. behavioural variant frontotemporal dementia, Alzheimer’s disease etc. according to consensus diagnostic criteria), gender, age at sample, age at onset of disease, weight, and the genotype of two polymorphisms previously shown to potentially affect PGRN concentration: *TMEM106B *rs1990622 and *GRN *rs5848. Authors who did not initially respond were contacted one further time. Data that was collected, including the institute, country, assay type and sample type, alongside the number of data points provided, are listed in Supplementary Table 1.

### Statistics

All statistical analysis was performed using GraphPad Prism (version 9.2.0), and data were tested for normality using the Shapiro Wilk test. Depending on normality, group differences were analysed using either a two tailed t-test or Mann Whitney test with *p* < 0.05 considered significant. Similarly, Pearson or Spearman correlations were calculated depending on normality. A ROC curve analysis was used for analysing clinical cut-off values with the maximum Youden’s index used to establish the best cut-off value.

## Results

In total, data were shared from 75 publications, consisting of PGRN concentrations from 7071 individuals (Table 1, Supplementary Fig. 1). Of these, 56 used the Adipogen assay (Adipogen Inc., Seoul, Korea) and 19 used other assays (5 used A&G pharmaceutical (Columbia, MD), 1 used BioVendor (Brno, Czech Republic), 2 used Mediagnost (Reutlingen, Germany) and 11 used R&D systems (Minneapolis MN, USA)). We therefore decided to focus on the Adipogen assay data as there were limited data on each of the other individual assays. Of the 56 studies, 38 investigated plasma, 12 serum, and 12 CSF concentrations of PGRN (with some papers studying more than one sample type).

**Table 1:** PGRN concentration data shared by assay and biofluid type. Total numbers are shown with available data on *GRN* mutation carriers shown in parentheses

	**A&G**	**Adipogen**	**BioVendor**	**Mediagnost**	**R&D**	**Others**	**Total**
Total	149 (7)	5058 (564)	56 (38)	55 (0)	1481 (6)	272 (1)	7071 (616)
Plasma	0 (0)	3301 (438)	0 (0)	0 (0)	671 (0)	147 (1)	3986 (439)
Serum	149 (7)	758 (125)	53 (35)	49 (0)	649 (6)	0 (0)	1658 (173)
CSF	0 (0)	1346 (19)	32 (23)	55 (0)	0 (0)	125 (0)	1558 (42)

### PGRN concentrations in people with GRN variants

We initially examined concentrations in people with variants in the *GRN* gene. We considered different variants including those with nonsense, frameshift, or deletion mutations as well as splice site mutations. Missense variants were divided into mutations in the signal peptide and variants after the signal peptide (termed ‘other missense mutations’).

### Plasma

**Fig. 1:**
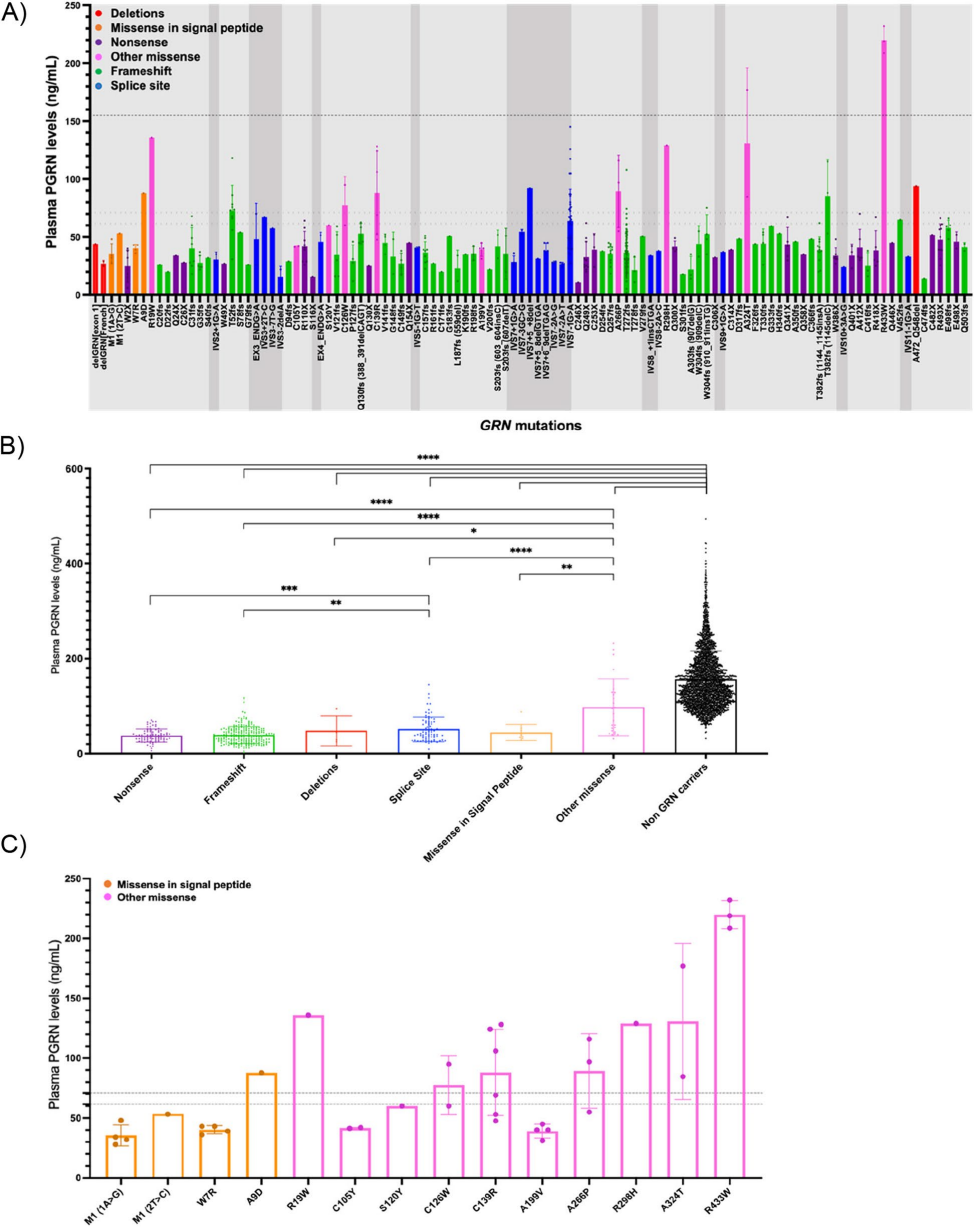
**A** Plasma PGRN concentrations across individual *GRN* variants. Light grey shading denotes exonic regions and darker grey shading intronic regions. Dotted lines denote previously published with cut-offs for pathogenicity 61.55 [14] and 71.00 [30] and average non-GRN plasma PGRN concentration (156.02 ng/mL). Different colours represent different types of variants. **B** Plasma PGRN concentrations by mutation type. * *P* < 0.05, ** *P* < 0.01, *** *P* < 0.001, **** *P* < 0.0001, two-tailed Mann–Whitney Test. Small sample sizes in deletions (*n* = 4) and missense in signal peptide (*n* = 10). **C** Plasma PGRN concentrations in different *GRN* missense variants. Dotted lines denote cut-offs previously published of 61.55 [14] and 71.00 [30]. Error bars indicate standard error of the mean (SEM)

Data were available on plasma PGRN concentration using the Adipogen assay in 3301 individuals (Table 1). Concentrations were variable both between and within individual *GRN* variants (Fig. 1A). Grouping these variants by type of mutation (Fig. 1B), the other missense mutations had significantly higher plasma PGRN concentrations compared with all other mutation groups (versus missense in the signal peptide (*p* = 0.002), splice site (*p* < 0.0001), deletions (*p* = 0.042), frameshift (*p* < 0.0001) and nonsense mutations (*p* < 0.0001)). Significantly higher levels were also observed in the splice site group compared with both frameshift and nonsense mutations (*p* < 0.0001 and *p* = 0.001, respectively).

Focussing on missense mutations (Fig. 3), plasma PGRN levels were generally in the normal range for all mutations in the “other missense” group (i.e. those after the signal peptide). However, two mutations, C105Y and A199V*,* were exceptions to this, yielding low plasma PGRN levels (below both previously defined cut-offs of 61.5 ng/mL [14] and 71.0 ng/mL [30] in all measured cases. Interestingly, in some of the other missense mutations levels were variable, with concentrations both below and above the previously defined cut-offs (e.g. C139R, A266P).

**Fig. 2:**
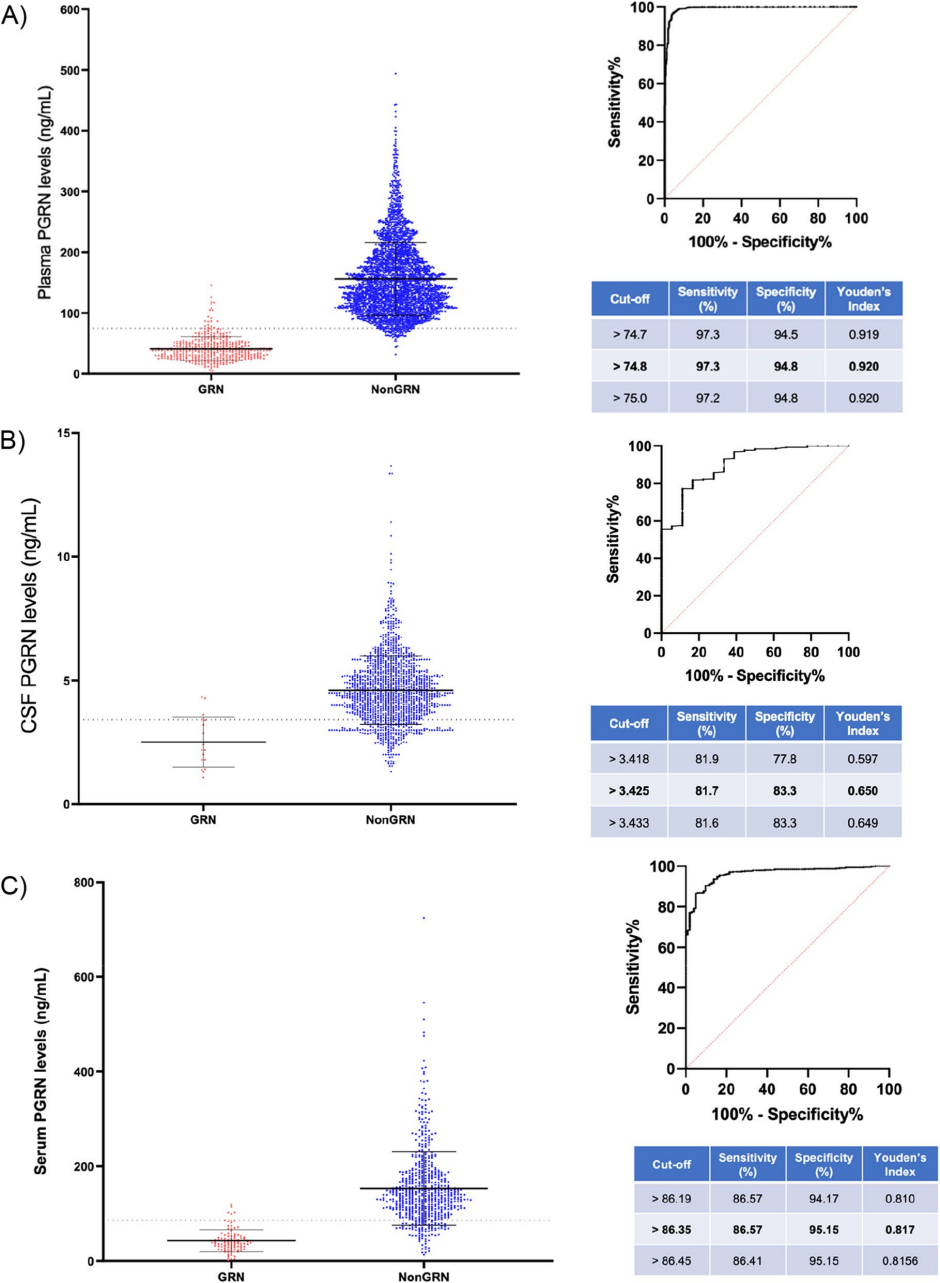
**A** PGRN plasma concentrations in *GRN* mutation carriers (GRN) and non-mutation carriers (Non-GRN). Cut-off determined using the optimal Youden’s index. **B** PGRN CSF concentrations in *GRN* mutation carriers (GRN) and non-mutation carriers (non-GRN). Cut-off determined using the optimal Youden’s index. **C** Serum PGRN concentrations in both GRN mutation carriers (GRN) and non-mutation carriers (non-GRN). Cut-off determined using the optimal Youden’s index. Error bars indicate standard error of the mean (SEM)

Using this large dataset of plasma PGRN concentrations, a cut-off for *GRN* mutation pathogenicity was established as 74.8 ng/mL with a Youden’s index of 0.92 (sensitivity 97.3; specificity 94.8), based on 3265 individuals (401 *GRN* mutation carriers (excluding other missense mutations) and 2864 non-carriers (both healthy and disease controls)) (Fig. 2A).

### Serum

Less data were available for serum concentrations using the Adipogen assay, with measures available for 758 individuals. A cut-off for *GRN* mutation pathogenicity was established as 86.3 ng/mL with a Youden’s index of 0.82, based on 125 *GRN* mutation carriers and 633 non-carriers (Fig. 2C). Serum levels across all cases (mutation carriers and non-carriers) showed a trend for correlation with plasma levels, *r* = 0.67, *p* = 0.0696 (Supplementary Fig. 3).

### CSF (Fig. 2B)

CSF PGRN concentrations using the Adipogen assay were available in 1346 individuals. A cut-off for *GRN* mutation pathogenicity was established as 3.43 ng/mL with a Youden’s index of 0.65, based on 19 *GRN* mutation carriers and 1327 non-carriers (Fig. 2B). CSF levels correlated with plasma levels, *r* = 0.33, *p* < 0.001 but only showed a trend to correlation with serum concentrations, *r* = 0.15, *p* = 0.0780 (Supplementary Fig. 3).

### PGRN concentrations by clinical phenotype

#### Neurodegenerative disorders

In the *GRN* mutation carrier group, no differences were observed in plasma PGRN concentrations between patients with a diagnosis of behavioural variant FTD (bvFTD) and those with a primary progressive aphasia (PPA) (Supplementary Fig. 4).

**Fig. 3:**
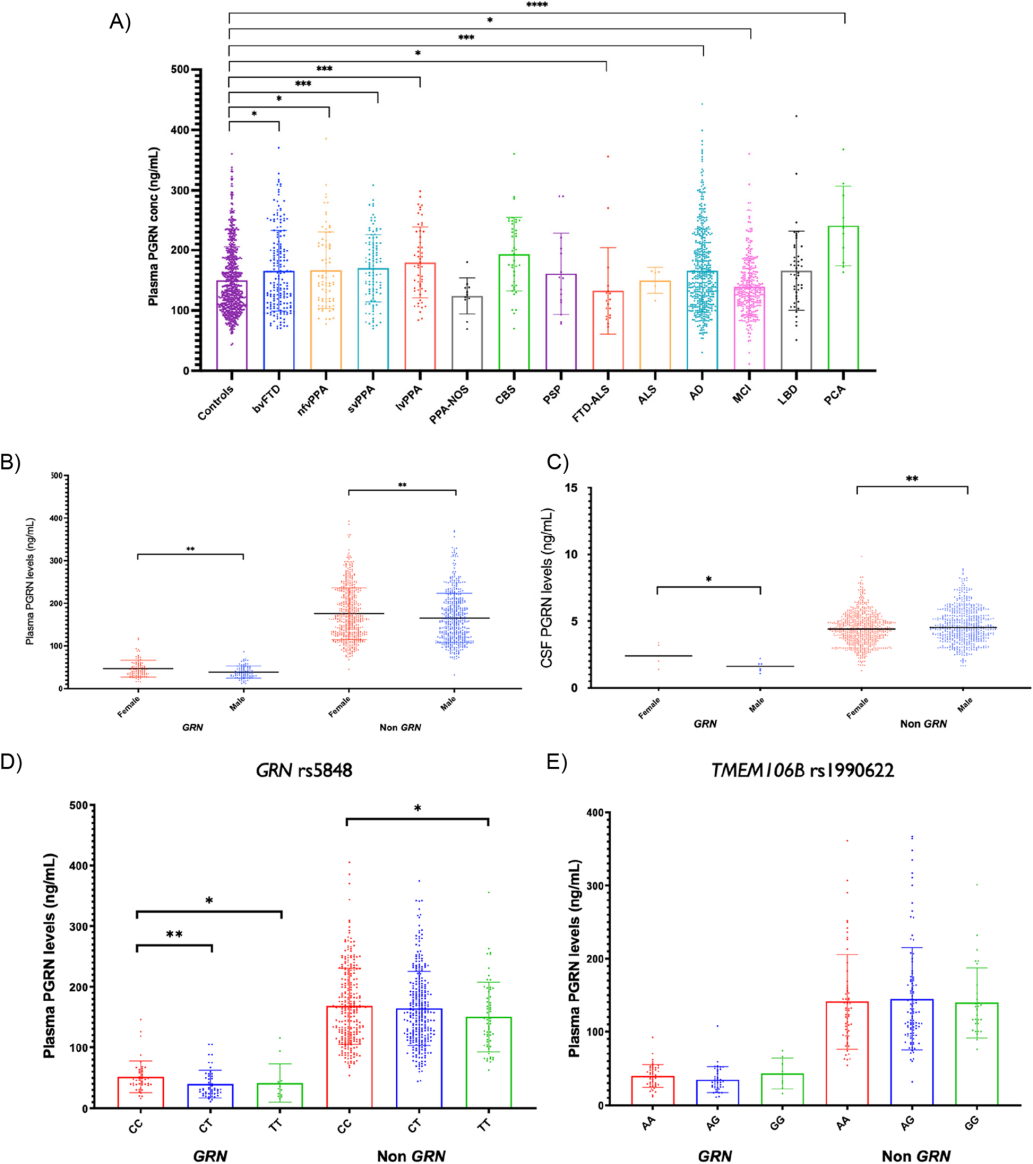
**A** Plasma PGRN concentrations across different clinical diagnoses. * *P* < 0.05, ** *P* < 0.01, *** *P* < 0.001, **** *P* < 0.0001, two-tailed Mann–Whitney Test. Significant differences compared with controls are shown on the graph. Additionally, PGRN concentrations were significantly different for bvFTD v CBS (**), FTD-ALS (*), MCI (***) and PCA (**); lvPPA v PPA-NOS (**), FTD-ALS (***), MCI (****) and PCA (*); nfvPPA v CBS (*), FTD-ALS (***), MCI (**) and PCA (**); svPPA v PPA-NOS(**), CBS (*), FTD-ALS(**), MCI (****) and PCA (**); PPA-NOS v FTD-ALS (***), AD (*), LBD (*) and PCA (****); CBS v AD (**), LBD (*) and MCI (****); PSP v PCA (**); FTD-ALS v CBS (***), AD (**), LBD (**) and PCA (***); ALS v PCA (**); AD v MCI (****) and PCA (***); MCI v LBD (**) and PCA (****); LBD v PCA (**). (bvFTD: behavioural variant FTD, PPA: primary progressive aphasia, nfvPPA: nonfluent variant PPA, svPPA: semantic variant PPA, lvPPA: logopenic variant PPA, FTD-ALS: frontotemporal dementia—amyotrophic lateral sclerosis, ALS: amyotrophic lateral sclerosis, CBS: corticobasal syndrome, PSP: progressive supranuclear palsy, AD: Alzheimer’s disease, MCI: mild cognitive impairment, LBD: lewy body disease, PCA: posterior cortical atrophy). Small sample sizes in ALS (*n* = 1), PCA (*n* = 10), PPA-NOS (*n* = 11), FTD-ALS (*n* = 18), PSP (*n* = 16). **B** Plasma and **C** CSF PGRN concentrations in females and males in both *GRN* mutation carriers (*GRN*) and non-mutation carriers (non-GRN). * *P* < 0.05, ** *P* < 0.01, two-tailed Mann–Whitney Test. **D** Plasma PGRN concentrations in those with different *GRN* rs5848 and **E** *TMEM106b* rs1990622 polymorphisms in both GRN mutation carriers (GRN) and non-mutation carriers (non-GRN). * *P* < 0.05, ** *P* < 0.01, two-tailed Mann–Whitney Test. Error bars indicate standard error of the mean (SEM)

In those *without GRN* mutations, plasma PGRN concentrations were significantly *higher* than controls in multiple groups (Fig. 3A), including not only those with FTD syndromes, but also those with typical and atypical forms of Alzheimer’s disease (including posterior cortical atrophy).

PGRN concentrations were also available for a number of non-neurodegenerative conditions, with lower plasma PGRN levels seen in bipolar disorder compared with controls (*p* = 0.018), but not in diabetes (*p* = 0.543) (Supplementary Fig. 5A). Serum PGRN levels were found to be lower in Gaucher’s disease compared with controls (*p* < 0.001) but concentrations in both osteoarthritis and rheumatoid arthritis groups were higher (both *p* < 0.001) (Supplementary Fig. 5B).

### PGRN concentration differences by sex

Plasma PGRN concentrations were significantly higher in women compared to men in *GRN* mutation carriers (mean (standard deviation) 46.6 (19.6) ng/mL vs (38.9 (14.2) ng/mL, *p* = 0.007), as well as in non-carriers (175.7 (60.7) ng/mL vs (165.7 (58.1) ng/mL *p* = 0.006, Fig. 3B). CSF PGRN levels were also significantly higher in women (2.5 (0.7) ng/mL) compared to men (1.6 (0.4) ng/mL) in the *GRN* mutation carrier group (*p* = 0.037), but not in the non-carrier group, where the opposite result was seen (men 4.7 (1.3) ng/mL, women 4.5 (1.2) ng/mL, *p* = 0.003) (Fig. 3C).

### PGRN concentration with increasing age

Plasma PGRN concentrations showed a weak but significant positive correlation with age at sampling (*r* = 0.09, *p* < 0.0001) in non-carriers with a similar correlation seen in *GRN* mutation carriers (*r* = 0.15, *p* = 0.0031) (Supplementary Fig. 6).

There was no significant correlation of plasma PGRN concentration with age at symptom onset in *GRN* mutation carriers. However, there was a significant positive correlation in an FTD cohort without mutations, *r* = 0.18, *p* < 0.0001. In contrast, there was a significant negative correlation in those with Alzheimer’s disease (AD), *r* = -0.24, *p* < 0.0001 i.e. lower concentrations associated with older age at onset (Supplementary Fig. 7).

### PGRN concentration by weight

No significant correlations were seen between plasma PGRN levels and weight in either *GRN* mutation carriers (*r* = -0.08, *p* = 0.7354) or in those without mutations (*r* = -0.07, *p* = 0.0751) (Supplementary Fig. 8).

### PGRN concentration in relation to the GRN rs5848 and TMEM106b rs1990622 polymorphisms

Significantly higher plasma PGRN levels were seen in those with the *GRN rs5848* CC genotype compared to those with both the CT and TT genotypes in *GRN* mutation carriers (*p* = 0.003 and *p* = 0.027 respectively; note that for heterozygous individuals these analyses did not take into account which rs5848-allele produced PGRN. Significantly higher concentrations were also seen in non-mutation carriers with the CC genotype compared with those with TT (*p* = 0.041) (Fig. 3D).

We found no significant differences between concentrations in those with the AA, AG or GG *TMEM106B *rs1990622 genotypes, either in *GRN* mutation carriers or non-carriers (Fig. 3E).

## Discussion

This study has highlighted a number of important factors that influence biofluid PGRN concentrations across a large cohort of individuals including type of *GRN* variant, clinical phenotype, age at sampling and at symptom onset, sex and the *GRN* rs5848 polymorphism.

Firstly, we highlighted the variability of plasma PGRN levels across *GRN* mutation types, most notably showing levels for missense mutations after the signal peptide to be significantly higher than the other mutation groups, suggesting that the majority of these mutations may not be pathogenic but more likely either risk factors for FTD or just benign polymorphisms. This is supported by earlier work indicating missense mutations yield PGRN levels higher than other *GRN* mutation carriers [9]. However, this was not true for all mutations, as two mutations in this group, C105Y and A199V led to pathogenic levels of plasma PGRN. Previous functional studies of these mutations have shown defects in RNA splicing, PGRN secretion and proteolytic processing likely to cause a pathological effect (The A199V is in fact two nucleotides away from the splice site and thus is technically a splice-site mutation) [22, 20, 24]. This supports the suggestion that these two missense mutations are pathogenic. Furthermore, as suspected, the missense mutations in the signal peptide generally yielded low, likely pathogenic, levels of plasma PGRN which is corroborated by existing reports of disrupted protein interactions and low PGRN levels in these mutations [28, 29]. These findings provide a key insight into the potential pathogenicity of certain *GRN* mutations and may help identify suspected pathogenic mutations in the future. However, what these studies do not tell us about is whether missense mutations cause abnormal functioning progranulin in the presence of normal PGRN concentrations in biofluids, and further work is required to understand the complex downstream pathways that might lead to neurodegeneration in these cases.

Secondly, we established a novel cut-off value for *GRN* mutation pathogenicity at a plasma PGRN level of 74.8 ng/mL based on data from 3265 individuals (excluding individuals with ‘other missense mutations’). This newly calculated cut-off is very close to one initially reported in the literature (74.4 ng/ml) [13]. Other previously established cut-offs were generally lower than this value, at 61.5 ng/mL and 71.0 ng/mL [14, 30] although an early study reported a much higher cut-off of 112.0 ng/mL [9]. This variation could be due to the inclusion criteria, with different populations, smaller sample sizes in some studies, and in some cases a different set of mutation types. There may also have been pre-analytical or analytical differences between centres which we are unaware of. Lastly, the control group of ‘non-carriers’ may have varied between studies. The cut-off generated here is based on the largest sample size reported to our knowledge, incorporating different mutation types and a spread of populations. However, as seen with previous studies, overlap in plasma PGRN levels between mutation carriers and non-carriers is still observed (i.e. there is no absolute cut-off), likely reflecting the influence of other biological factors on these levels such as hormonal or metabolic disorders.

We also identified cut-offs for CSF and serum PGRN levels in this cohort, which could prove beneficial for a more complete picture of how PGRN levels are analysed. Notably however, the lack of tight correlations between different fluid PGRN measures found here could suggest differential PGRN expression throughout the body. This is supported by prior findings of significantly lower CSF PGRN levels compared with serum levels in some *GRN* mutation carriers and other reports proposing differentially derived PGRN in CSF and blood [8, 26, 34].

Although *GRN* mutation carriers are associated with lower PGRN concentrations, we found that across multiple neurodegenerative diseases PGRN levels were generally higher than a control population e.g. in AD and non-*GRN*-FTD, as reported in a previous multicentre Italian study [14]. Numerous reviews have highlighted PGRN’s key role in neurodegeneration [5, 19, 27], although other studies have not reported differences in plasma PGRN levels in AD despite a reported increase in PGRN mRNA [6, 15]. Similarly, previous research has suggested that people with FTD without *GRN* mutations have similar levels to controls [15, 30]. Further work is needed to better understand the role of increased PGRN in these disorders.

We also analysed whether PGRN differences were observed in non-neurodegenerative diseases, finding that certain conditions, such as bipolar disorder and Gaucher disease, were linked to lower PGRN concentrations whereas others, such as arthritis, had higher biofluid PGRN concentration. This mirrors findings reported previously for these conditions, emphasising the multitude of processes PGRN is involved in and the need to consider comorbidities when interpreting levels [1, 3, 11, 18, 21]. For example, PGRN concentrations may be more challenging to interpret in the differential diagnosis of people with FTD compared with bipolar disorder [12, 21].

Sex differences have been previously reported in FTD [17] including PGRN levels across different biofluids. Nicholson and colleagues investigated the differential regulation between CSF and plasma PGRN, suggesting that this is linked to biological sex, with plasma PGRN levels higher in women and CSF PGRN levels higher in men [26]. This is consistent with our findings on sex differences in this cohort as well as other prior studies, with female plasma PGRN levels higher than males [30]. We also observed higher CSF PGRN levels in men in the non-carrier group, supporting the hypothesis of Nicholson et al. but notably found the opposite pattern in *GRN* mutation carriers, who yielded higher CSF PGRN levels in females. This potentially indicates a *GRN* mutation specific sex difference in PGRN activity in the central nervous system. Certainly, sex differences appear to be important in normal PGRN function, with only female *GRN* mutation mice developing bone formation defects compared with wildtype, and *GRN* expression linked to oestrogen activity during brain development [32, 33]. Collectively, these findings reveal the complexity of PGRN activity both peripherally and centrally and highlight the effect of biological sex on the function of this protein, something which should be considered for upcoming therapeutic trials.

A previous multicentre Italian study reported that the lowest plasma PGRN levels in *GRN* mutation carriers were associated with an anticipation of disease onset of about 9 years [14], although a further study did not find any correlation with either age at onset or age at sample [25]. In the present study, we found a significant positive correlation between age at symptom onset and plasma PGRN level in people with FTD without *GRN* mutations, where an earlier onset is seen in those with lower PGRN levels. However, we also saw a significant positive correlation between age at sampling and PGRN level in these non-carriers, which could be influencing our results, indicating a general increase in PGRN level with age. The reason for this increase is unclear although may be due to inflammatory changes with increasing age. In contrast, when analysing correlations in people with AD, we saw a significant negative correlation between plasma PGRN level and age at onset, indicating that higher levels of PGRN are linked to earlier ages of onset. Interestingly, Suarez-Calvet and colleagues report an increase in CSF PGRN level as the disease course progresses supporting the idea that PGRN plays a role in the pathogenicity of this condition [31], although the exact relationship between AD and PGRN requires further study.

We additionally found that the *GRN *rs5848 polymorphism influences plasma PGRN levels in both *GRN* mutation carriers and non-carriers, with the lowest levels in those with the TT genotype. This finding was previously reported by Hsiung and colleagues [16], who reported reduced serum PGRN levels for this genotype and speculated that this was related to miR-659 dependent translational inhibition. This was also highlighted in work by Nicholson and colleagues [26] who demonstrated a significant association between rs5848 and CSF PGRN concentration. Additionally, it has been previously reported that *rs5848* significantly affects GRN mRNA levels both centrally and peripherally and has been linked to both AD and Parkinson’s disease [4, 23]. Together, these findings suggest rs5848 is a key influencer of PGRN levels and could also help understand the role of PGRN in other neurodegenerative diseases. Interestingly, the *TMEM106b *rs1990622 polymorphism was not associated with differences in plasma PGRN levels in this large cohort. This finding suggests that the influence of this well-known risk factor for GRN-associated FTD is unlikely to be modulated through PGRN levels, as speculated previously [10]. However, more research is required to understand this fully.

Lastly, it is important to note the limitations of this study. One reason for variation in PGRN levels between studies included here is that despite testing the same fluid type with the same assay, the tests are performed by different researchers in different laboratories with different assay batches. Secondly, although the numbers of cases with available concentrations to study was large, once stratified, numbers for individual comparisons were often much smaller. Thirdly, for many of the conditions studied (including AD), we did not have access to robust disease severity measures. Finally, it should also be noted that in the absence of a certified reference material and a value assigned for PGRN concentration by a certified reference method, different assays are not standardized to each other; hence, laboratory- and assay-specific validation of the cut-points reported here remains important.

In summary, this large and diverse cohort of PGRN levels has allowed us to firstly refine the plasma PGRN cut-off level to predict GRN mutations, secondly, confirm the differential impact of the mutation type on blood PGRN levels, thirdly, highlight the high variability across missense mutations in *GRN*, and lastly, highlight the numerous factors which influence PGRN biofluid levels both GRN mutation carriers and non-carriers (i.e. clinical diagnosis, sex, age, GRN rs5848 genotype). These factors should be considered when utilising this marker, generating a more personalised approach to treatment. These results also reflect the need for the identification of additional factors which affect PGRN biofluids levels and thus hopefully modulate disease onset and/or progression, providing a more comprehensive picture of this disease as we continue into the era of therapeutic trials.

### Supplementary Information

**Additional file 1:** **Supplementary Figure 1.** Data collection flowchart. **Supplementary Figure 2.** Serum PGRN concentrations in both GRN mutation carriers (GRN) and non-mutation carriers (non-GRN). Cut-off determined using the optimal Youden’s index. **Supplementary Figure 3.** A) Correlation between serum and plasma PGRN levels in this cohort (*p*= 0.0696). B) Correlation between CSF and serum PGRN levels in this cohort (*p* = 0.0780). C) Correlation between CSF and plasma in this cohort (*p*<0.0001). **Supplementary Figure 4.** No significant difference in plasma PGRN levels between bvFTD (behavioural variant FTD) and PPA (primary progressive aphasia)* GRN* mutation carriers.**Supplementary Figure 5.** A) Correlation between plasma PGRN levels and age at sampling in *GRN* mutation carriers. B) Correlation between plasma PGRN levels and age at sampling in non *GRN* mutation carriers. ** *P* < 0.01,**** *P* < 0.0001, Spearman correlation. **Supplementary Figure 6.** A) Correlation between plasma PGRN levels and age of onset in *GRN* mutation carriers. B) Correlation between plasma PGRN levels and age of onset in non-*GRN* mutation carriers. C) Correlation between plasma PGRN levels and age of onset in people with AD. **** *P* < 0.0001, Spearman correlation. **Supplementary Figure 7.** A) Correlation between plasma PGRN levels and weight in *GRN* mutation carriers. B) Correlation between plasma PGRN levels and weight in non *GRN* mutation carriers. **Supplementary Figure 8.** A) Differences in plasma PGRN levels measured with the Adipogen assay between clinical diagnoses in this cohort. B) Differences in serum PGRN levels measured with the Adipogen assay between clinical diagnoses in this cohort. * *P* < 0.05, ** *P* < 0.01, *** *P* < 0.001, **** *P* < 0.0001, two-tailed Mann-Whitney Test. **Supplementary Table 1.** List of institutions, countries, assay types and sample types included in this dataset alongside the number of data point provided.

## Data Availability

The datasets used and/or analysed during the current study are available from the corresponding author on reasonable request.
