# Supplementary material for: A systematic review of progranulin concentrations in biofluids in over 7,000 people—assessing the pathogenicity of GRN mutations and other influencing factors
Source: Alzheimers Res Ther. 2024 Mar 28;16:66. doi: 10.1186/s13195-024-01420-z (PMC10976725; doi:10.1186/s13195-024-01420-z)
Supplement: Supplementary file 1 — Additional file 1: Supplementary Figure 1. Data collection flowchart. Supplementary Figure 2. Serum PGRN concentrations in both GRN mutation carriers (GRN) and non-mutation carriers (non-GRN). Cut-off determined using the optimal Youden’s index. Supplementary Figure 3. A) Correlation between serum and plasma PGRN levels in this cohort (p= 0.0696). B) Correlation between CSF and serum PGRN levels in this cohort (p = 0.0780). C) Correlation between CSF and plasma in this cohort (p<0.0001). Supplementary Figure 4. No significant difference in plasma PGRN levels between bvFTD (behavioural variant FTD) and PPA (primary progressive aphasia) GRN mutation carriers.Supplementary Figure 5. A) Correlation between plasma PGRN levels and age at sampling in GRN mutation carriers. B) Correlation between plasma PGRN levels and age at sampling in non GRN mutation carriers. ** P < 0.01,**** P < 0.0001, Spearman correlation. Supplementary Figure 6. A) Correlation between plasma PGRN levels and age of onset in GRN mutation carriers. B) Correlation between plasma PGRN levels and age of onset in non-GRN mutation carriers. C) Correlation between plasma PGRN levels and age of onset in people with AD. **** P < 0.0001, Spearman correlation. Supplementary Figure 7. A) Correlation between plasma PGRN levels and weight in GRN mutation carriers. B) Correlation between plasma PGRN levels and weight in non GRN mutation carriers. Supplementary Figure 8. A) Differences in plasma PGRN levels measured with the Adipogen assay between clinical diagnoses in this cohort. B) Differences in serum PGRN levels measured with the Adipogen assay between clinical diagnoses in this cohort. * P < 0.05, ** P < 0.01, *** P < 0.001, **** P < 0.0001, two-tailed Mann-Whitney Test. Supplementary Table 1. List of institutions, countries, assay types and sample types included in this dataset alongside the number of data point provided. [file 13195_2024_1420_MOESM1_ESM.docx]

**Appendix**

**Supplementary Figures and tables**

Supplementary Figure 1: Data collection flowchart


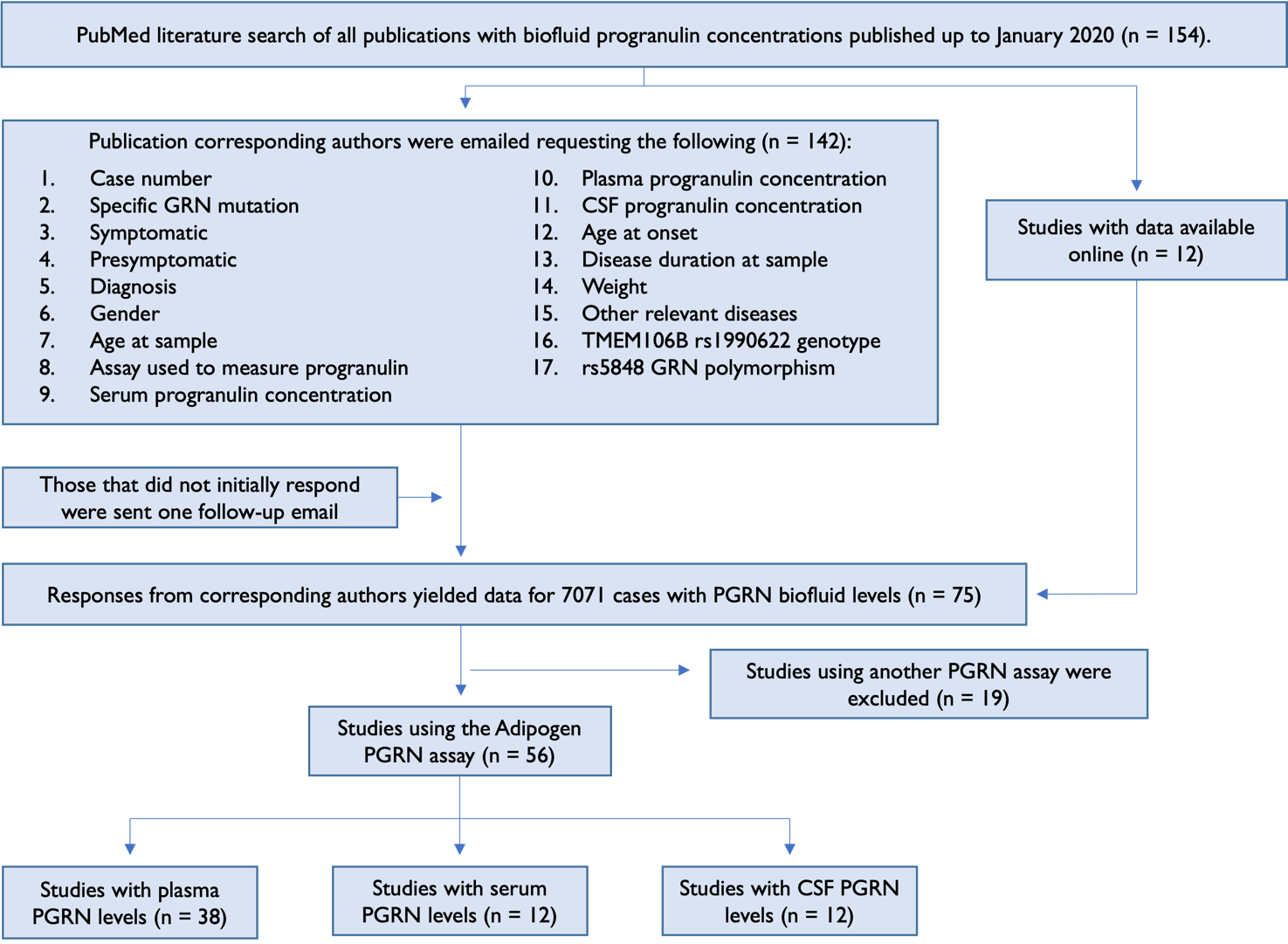


Supplementary Figure 3. A) Correlation between serum and plasma PGRN levels from the same individuals in this cohort (p = 0.0696). B) Correlation between CSF and serum PGRN levels from the same individuals in this cohort (p = 0.0780). C) Correlation between CSF and plasma from the same individuals in this cohort (p<0.0001).


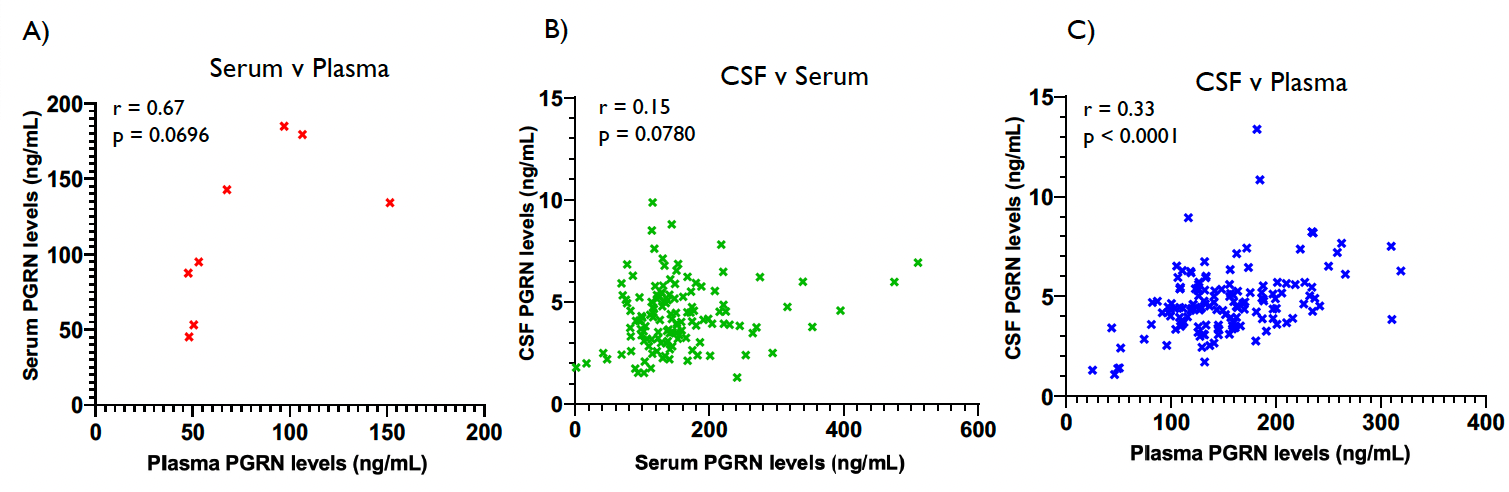


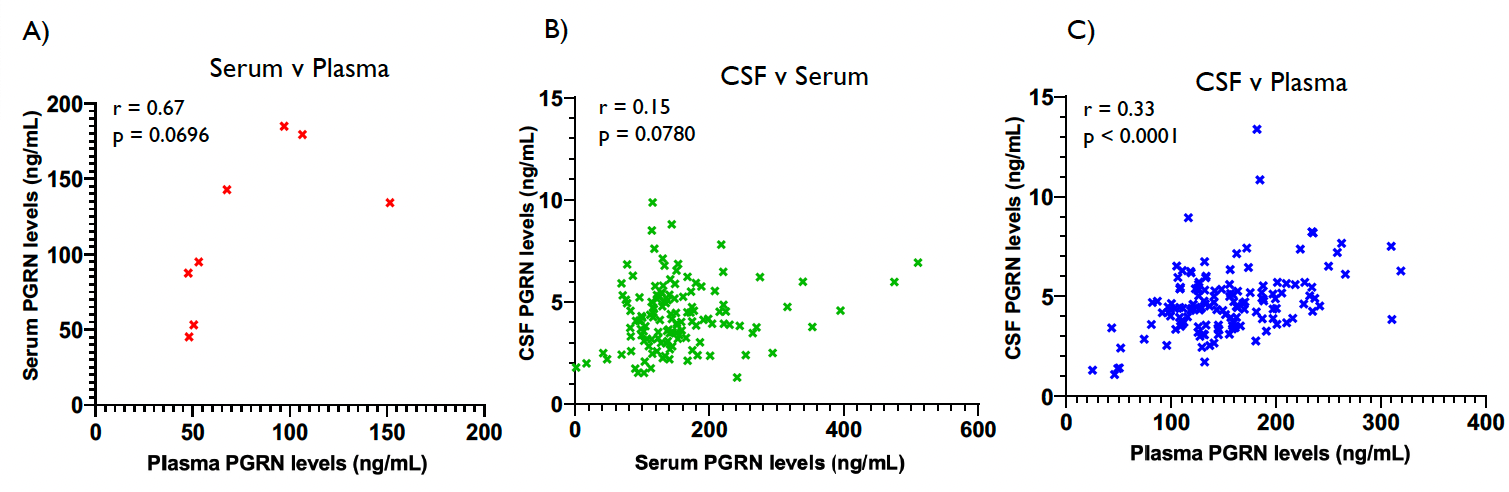

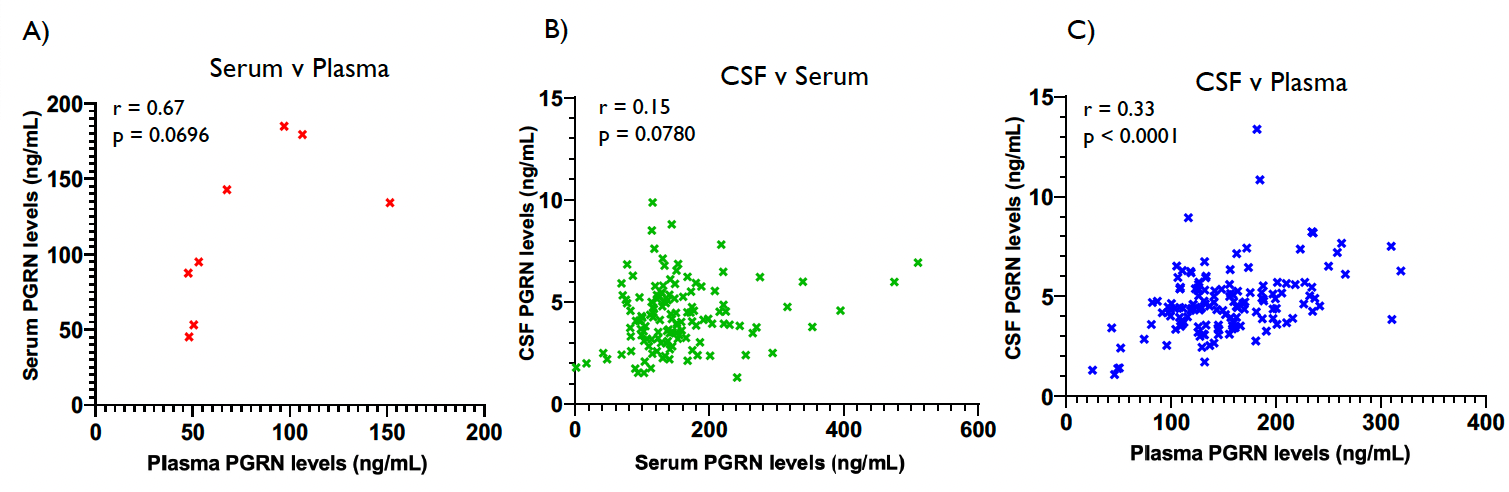


Supplementary Figure 4. No significant difference in plasma PGRN levels between bvFTD (behavioural variant FTD) and PPA (primary progressive aphasia) *GRN* mutation carriers. Error bars indicate standard error of the mean (SEM).

Supplementary Figure 5: A) Differences in plasma PGRN levels measured with the Adipogen assay between clinical diagnoses in this cohort. B) Differences in serum PGRN levels measured with the Adipogen assay between clinical diagnoses in this cohort. * P < 0.05, ** P < 0.01, *** P < 0.001, **** P < 0.0001, two-tailed Mann-Whitney Test. Error bars indicate standard error of the mean (SEM).


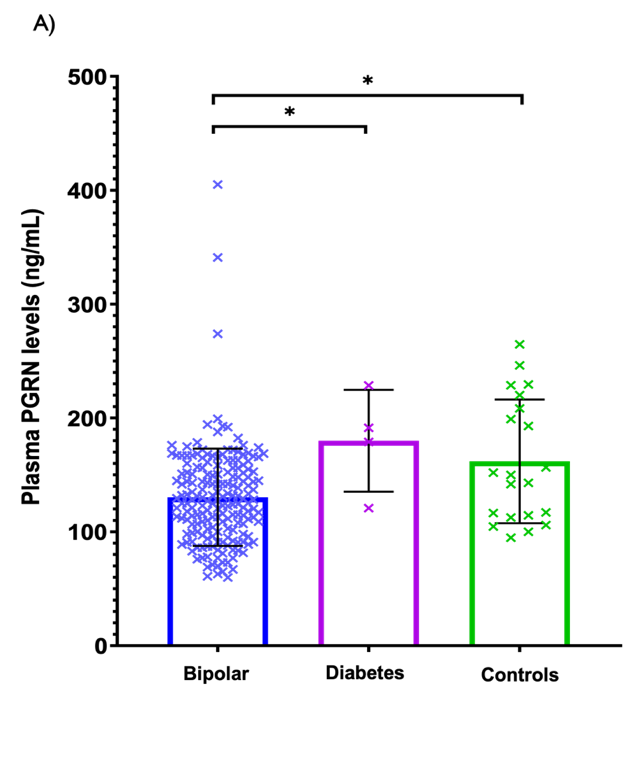

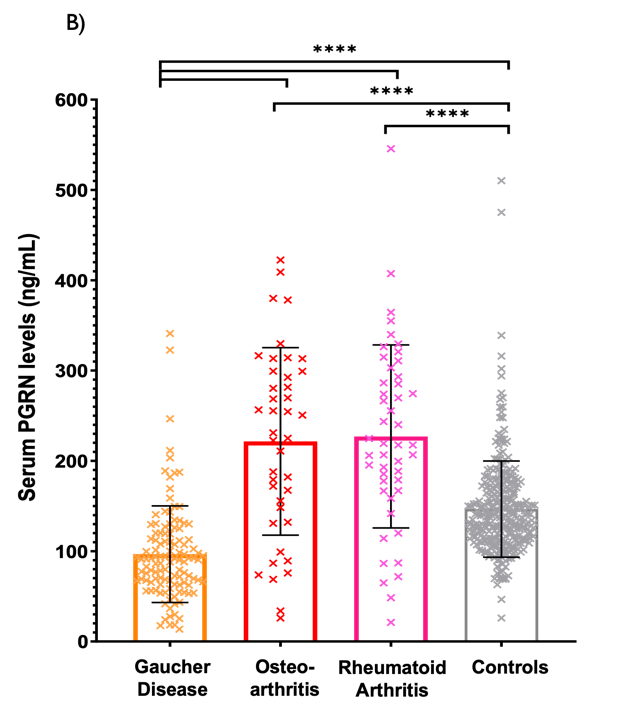


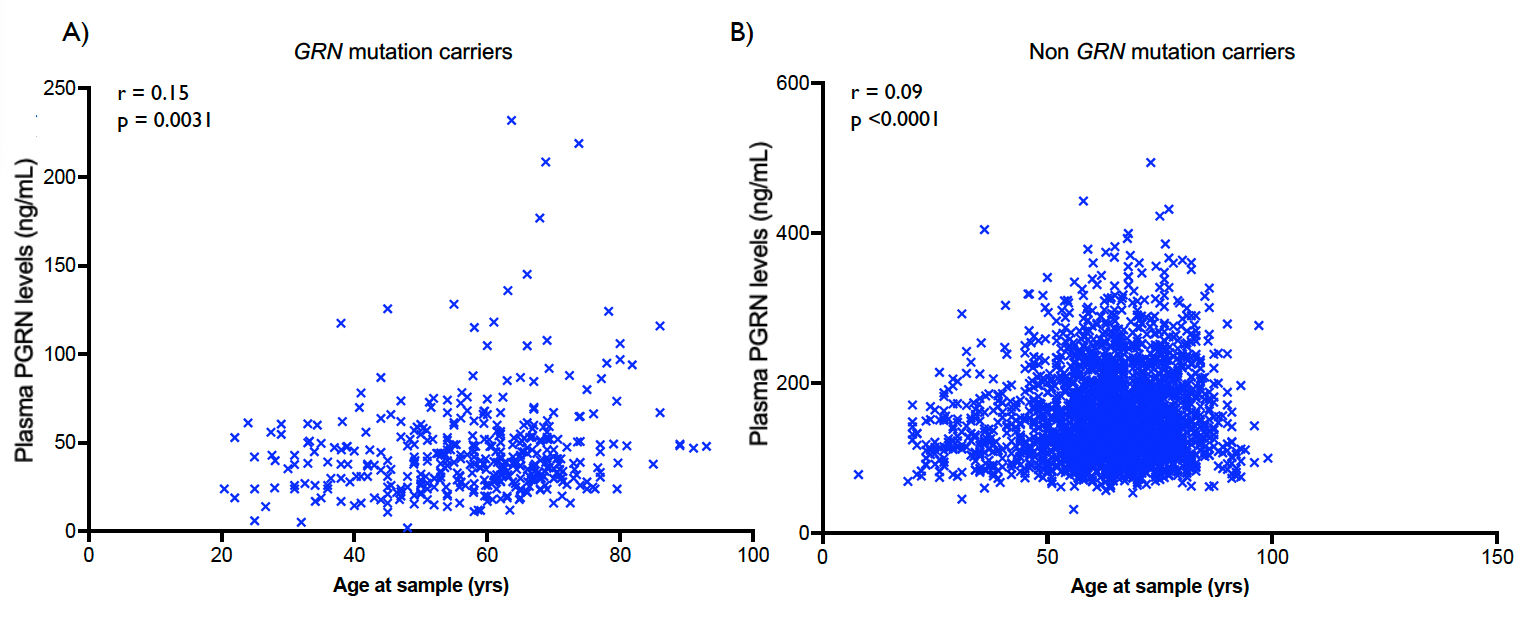
Supplementary Figure 6: A) Correlation between plasma PGRN levels and age at sampling in *GRN* mutation carriers. B) Correlation between plasma PGRN levels and age at sampling in non *GRN* mutation carriers. ** P < 0.01, **** P < 0.0001, Spearman correlation.


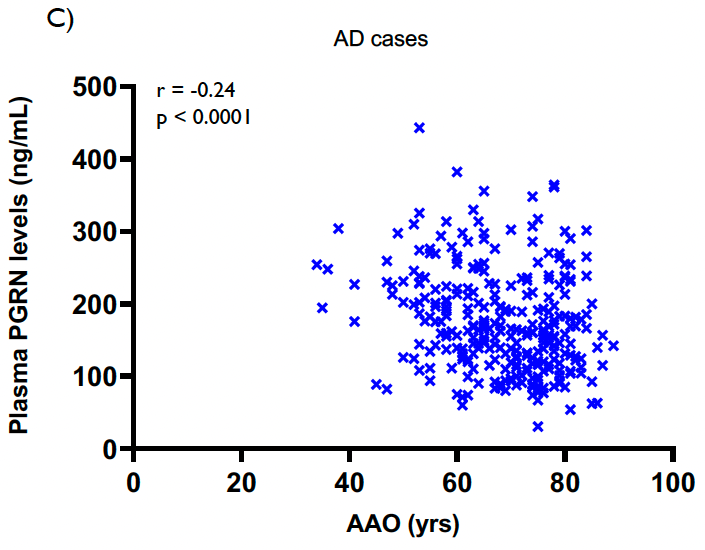
Supplementary Figure 7: A) Correlation between plasma PGRN levels and age of onset (AAO) in *GRN* mutation carriers. B) Correlation between plasma PGRN levels and AAO in FTD cases without *GRN* mutations. C) Correlation between plasma PGRN levels and AAO in people with AD. **** P < 0.0001, Spearman correlation.


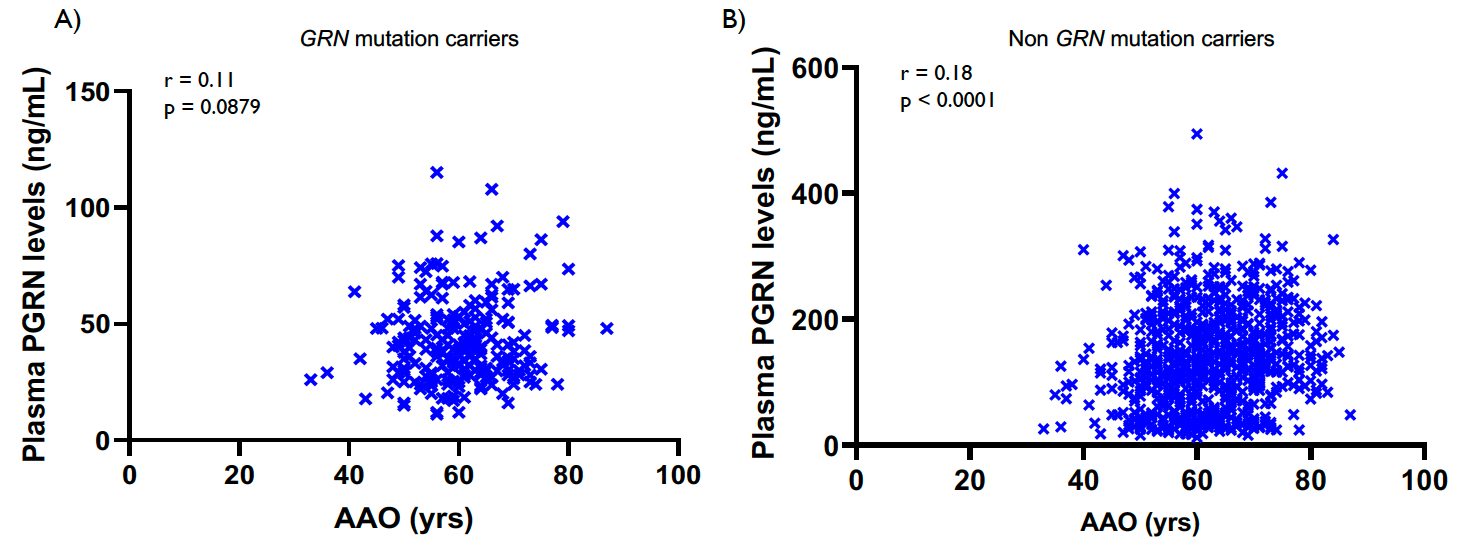


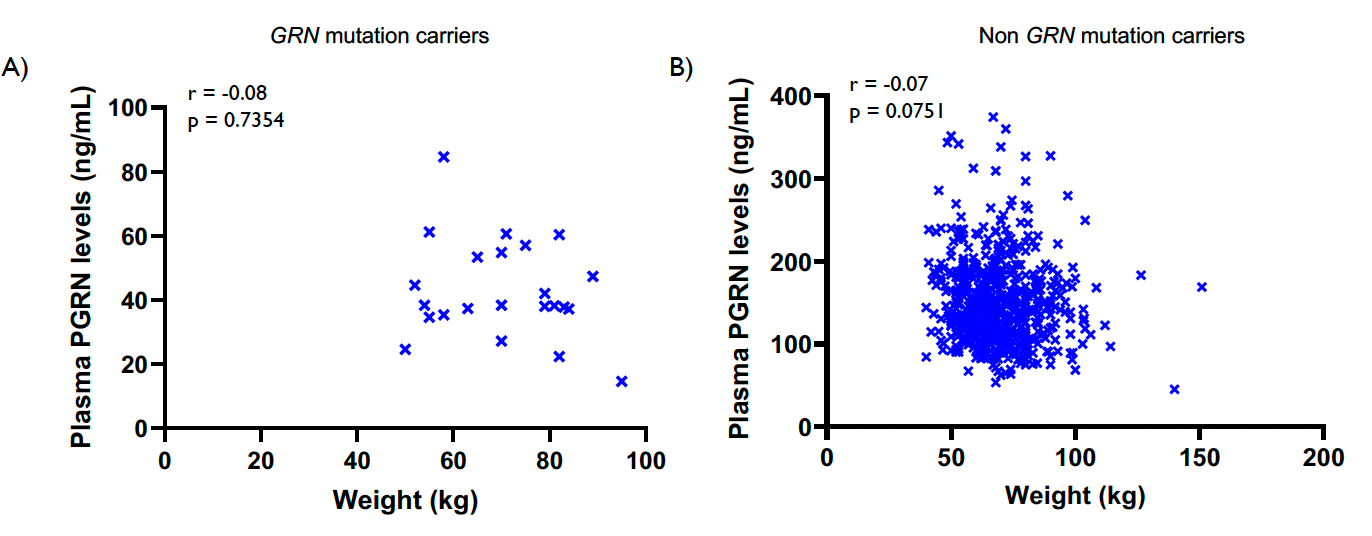
Supplementary Figure 8: A) Correlation between plasma PGRN levels and weight in *GRN* mutation carriers. B) Correlation between plasma PGRN levels and weight in non *GRN* mutation carriers.

Supplementary Table 1: List of institutions, countries, assay types and sample types included in this dataset alongside the number of data point provided.
